# The Role of Microbial Products in Green Enhanced Oil Recovery: Acetone and Butanone

**DOI:** 10.3390/polym13121946

**Published:** 2021-06-11

**Authors:** Bashirul Haq

**Affiliations:** Department of Petroleum Engineering, King Fahd University of Petroleum & Minerals, Dhahran 3126, Saudi Arabia; bhaq@kfupm.edu.sa or bhaq225@gmail.com

**Keywords:** microbial enhanced oil recovery (MEOR), green surfactant, green polymer, Green Enhanced Oil Recovery (GEOR)

## Abstract

Green enhanced oil recovery is an oil recovery process involving the injection of specific environmentally friendly fluids (liquid chemicals and gases) that effectively displace oil due to their ability to alter the properties of enhanced oil recovery. In the microbial enhanced oil recovery (MEOR) process, microbes produce products such as surfactants, polymers, ketones, alcohols, and gases. These products reduce interfacial tension and capillary force, increase viscosity and mobility, alter wettability, and boost oil production. The influence of ketones in green surfactant-polymer (SP) formulations is not yet well understood and requires further analysis. The work aims to examine acetone and butanone’s effectiveness in green SP formulations used in a sandstone reservoir. The manuscript consists of both laboratory experiments and simulations. The two microbial ketones examined in this work are acetone and butanone. A spinning drop tensiometer was utilized to determine the interfacial tension (IFT) values for the selected formulations. Viscosity and shear rate across a wide range of temperatures were measured via a Discovery hybrid rheometer. Two core flood experiments were then conducted using sandstone cores at reservoir temperature and pressure. The two formulations selected were an acetone and SP blend and a butanone and SP mixture. These were chosen based on their IFT reduction and viscosity enhancement capabilities for core flooding, both important in assessing a sandstone core’s oil recovery potential. In the first formulation, acetone was mixed with alkyl polyglucoside (APG), a non-ionic green surfactant, and the biopolymer Xanthan gum (XG). This formulation produced 32% tertiary oil in the sandstone core. In addition, the acetone and SP formulation was effective at recovering residual oil from the core. In the second formulation, butanone was blended with APG and XG; the formulation recovered about 25% residual oil from the sandstone core. A modified Eclipse simulator was utilized to simulate the acetone and SP core-flood experiment and examine the effects of surfactant adsorption on oil recovery. The simulated oil recovery curve matched well with the laboratory values. In the sensitivity analysis, it was found that oil recovery decreased as the adsorption values increased.

## 1. Introduction

**Figure 1:**
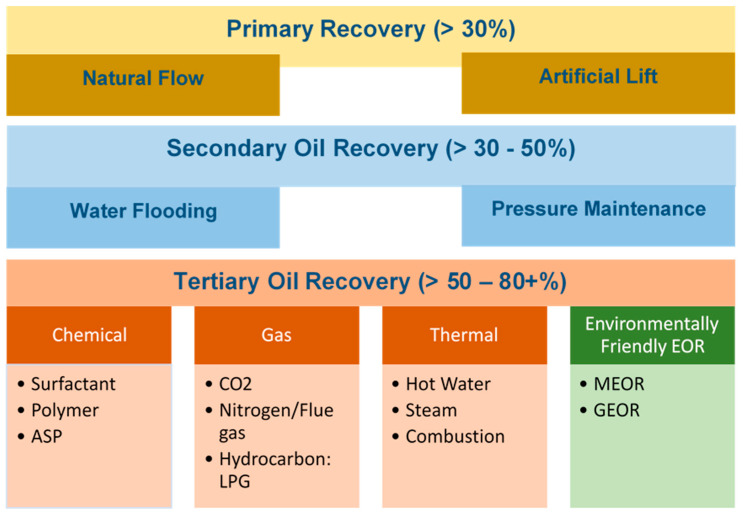
Classification of oil recovery processes.

Conventionally oil is recovered in three stages. These phases are primary, secondary, and tertiary. In the primary stage, around 30% of oil is produced from the reservoir due to natural drive mechanism and artificial lift. Secondary recovery starts when the primary method is no longer economically. The process involves waterflooding and pressure maintenance and produces about 30% to 50% oil. Finally, tertiary oil recovery, commonly known as enhanced oil recovery (EOR), uses gas, chemical and thermal energy to displace additional oil from the reservoir [1]. In this stage, injecting green chemicals such as surfactants and polymers, and microorganisms are used to extract extra oil. Green Enhanced Oil Recovery (GEOR) and MEOR are ex-situ and in-situ eco-friendly EOR process, respectively. In this process, environmentally friendly fluids are utilized to increase oil recovery. Oil recovery processes and the difference between Improved Oil Recovery (IOR) and EOR described in Figure 1.

Green enhanced oil recovery (GEOR) is an oil recovery process involving the injection of specific environmentally friendly fluids (liquid chemicals and gases) that effectively displace oil due to their ability to alter the properties of enhanced oil recovery (EOR). The main properties include phase behaviour, interfacial tension (IFT), wettability, mobility, capillary force, and viscosity. According to the Cambridge Dictionary, “environmentally friendly” is not harmful to the environment. The word “green” is also used in such a context, as are terms such as “environment-friendly,” “eco-friendly,” and “nature-friendly.” These words refer to goods and services, laws, guidelines, and policies that are negligibly harmful or do no harm to the environment. 

Microbes can produce in-situ bio-surfactants, polymers, alcohols (methanol, ethanol, and butanol), ketones (acetone and butanone), acids (formic acid, and acetic acid), and gases (methane, and carbon dioxide) in a reservoir. Haq [2] published a chapter called Green Enhanced Oil Recovery (GEOR) in the PhD Thesis title “The Role of Green Surfactants in microbial enhanced oil recovery”. After that, he started his journey to explore more about GEOR. In 2017, Haq et al [3]. published a paper titled “Green Enhanced Oil Recovery” in the APPEA Journal. Then, in 2019 [4,5,6], his group conducted several experiments to examine the combined effects of biosurfactants, APG, and butanol on recovering residual oil. The goal of their study was to develop biobased EOR processes. Such processes are an effective alternative to MEOR methods when MEOR is found to be slow and inefficient. The influence of microbial products: acetone, butanone, formic acid, acetic acid, CO_2_ and CH_4_ in GEOR are not studied. As a continuation of that work, acetone and butanone were selected for the present study to determine the effects of a green surfactant and polymer solution on recovering oil. The following paper will be discussed about the impact of acids in GEOR. 

Microbes produce products in the MEOR method, for example, surfactants, polymers, ketones, alcohols, and gases. These products reduce IFT and capillary force, increase viscosity and mobility, alter wettability, and boost oil production. In the last several years, microbial products such as bio-surfactants and alcohols have been studied to examine the potential of various EOR methods. A type of microbe called *Bacillus mojavensis* JF-2 has been used to produce a bio-surfactant called lipopeptide [7,8,9,10,11,12] at high temperatures and salinities in an anaerobic condition. This surfactant reduced the IFT from 29 to 0.01 dyne/cm between the oil and water. Mulligan et al. [13] found that the JF-2 bio-surfactant was anionic and similar to synthetic anionic surfactants. Several experiments were performed to determine the optimum IFT of the Bacillus mojavensis JF-2 bio-surfactant and 2,3-butanediol [9,10]. The optimum concentration of bio-surfactant was found to be between 50 and 60 ppm. Haq et al. [2,3,4,5,6] performed a series of experimental and simulation studies to explore the combined influence in EOR of microbial products, including bio-surfactants, alcohols, and green surfactants. In Haq et al. [3], combinations of JF2 bio-surfactant and butanol and the green surfactant alkyl polyglucoside (APG) and butanol were used to perform phase behaviour IFT measurement and core flooding experiments. It was reported that 0.50% APG, 0.5% to 1.00% butanol, and 2% NaCl gave a stable middle phase. A bio-surfactant of 45 mg/L (ppm) and 0.50% butanol with 2% NaCl produced about 2% incremental oil. The ionic effect and phase behaviour of a mixture of microbial butanol and APG was also investigated [6]. A 0.50% APG and 0.50% butanol solution had a minimal effect on temperature and salinity. In Haq et al. [6], 45 mg/L of JF2 bio-surfactant, 0.50% APG, and 0.50% butanol were flooded in a sandstone core at 52 °C and 1050 psi. This formulation was selected based on IFT and phase behaviour and gave 25% tertiary oil and 64% oil initial in place (OIIP). 

Surfactants’ recovery efficiency can be examined using core flood tests. However, conducting core flood experiments with a wide range of formulations is time-consuming and laborious. Reservoir simulation offers the ability to test different EOR processes under various conditions without requiring lengthy laboratory experiments. The combined effects of surfactants and alcohol on oil recovery efficiency and IFT and various concentration options must be added to the Eclipse black oil simulator to simulate MEOR and GEOR processes adequately. Haq et al. [6] modified the Eclipse simulator to add MEOR and GEOR options. The modified Eclipse simulator was then applied to simulate bio-surfactants and green core flood tests. 

It was determined that the microbial products of bio-surfactants and alcohol with green surfactants positively influenced GEOR. However, the impact of ketones on GEOR is not yet well understood and requires further study. Acetone is an organic compound and known as 2-propanone. The chemical structure of acetone is CH_3_-CO-CH_3_ and is a colourless liquid. It is mainly used in the chemical industry as a solvent and medicine as an antiseptic. Butanone is a colourless organic liquid. The IUPAC name of the compound is methyl ethyl ketone (MEK). The chemical formula is CH_3_-CO-CH_2_CH_3_. Butanone is commonly used as a solvent. The goal of the present work was to explore the influence of ketones on GEOR through experiments and simulation studies. 

## 2. Methodology and Material

### 2.1. Materials 

The APG 264 non-ionic surfactant was supplied by the BASF. The acetone, butanone, and Xanthan gum were purchased from Sigma Aldrich (Dammam, Saudi Arabia). The physicochemical properties of acetone and butanone are given in Table 1. The ketones were 99.98% pure. The NaCl was 99.87% pure and purchased from Sigma Aldrich. Arabian light crude oil was used throughout this research and supplied by Saudi Aramco (Dhahran, Saudi Arabia). The Arabian light oil had a degree API of 31.14 and a density of 0.87 g/cc when measured at a room temperature of 23 °C. The viscosity was 19.8 cp at 25 °C. 

Xanthan gum, the most common biopolymer, is commercially produced through the process of fermentation. It has a single glucuronic acid unit, two mannose units, and two glucose units of molar ratio 2.0, 2.0, and 2.8, respectively [14].

### 2.2. Selection of Green Surfactant and Concentration, and Microbial by-Products: Acetone and Butanone

Haq et al. [3] measured the IFT values of four types of APG surfactants at constant concentrations and salinities, keeping the pressure and temperature in the same condition. These were APG 264, APG 8166, APG 8105, and APG 8107. From this non-ionic group, APG 264 offered the best performance based on its IFT reduction capability. APG 264 was thus chosen for the core floods in the present research. Haq et al. [5] performed a series of IFT measurements to determine the optimum surfactant concentration for core flood experiments. The APG concentrations ranged from 0.20% to 2.00% with brine (2% NaCl). The optimum concentration selected was 0.50% for core flood experimentation. The same concentration was used in the present study. 

Microbes can produce in-situ bio-surfactants, polymers, alcohols, ketones, acids, and gases in a reservoir. Haq et al. [5] conducted several experiments to examine the combined effects of biosurfactants, APG, and butanol on recovering residual oil. The goal of their study was to develop biobased EOR processes. As a continuation of that work, acetone and butanone were selected for the present study to determine the effects of a green surfactant and polymer solution on recovering oil. 

### 2.3. Interfacial Tension (IFT) Measurement 

**Figure 2:**
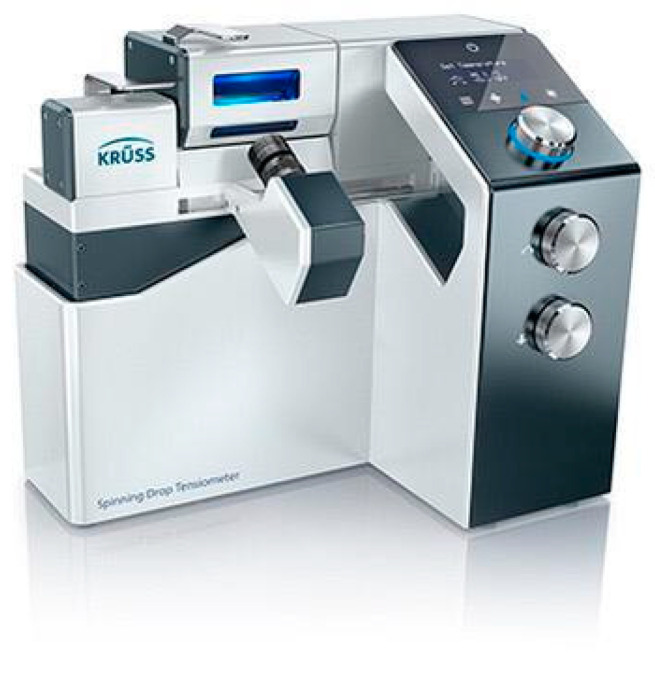
Spinning drop tensiometer.

The Spinning Drop Tensiometer (STD) 100 from KRUSS (Jeddah, Saudi Arabia) was used for the IFT measurements. The SDT 100 instrument is shown in Figure 2. The IFTs of the APG 264 and ketone mixtures were determined to identify the optimum concentration for the core flood experiments. It is necessary to measure the densities of both liquids: oil and brine. So, first, oil and sample densities were measured at 25 °C using a density meter. Then, the cell is cleaned using hot water and soap and calibrated using a sample with a known value. Next, the rotation speed is set to 2800 RPM. After that, the 20 mL sample is loaded, and the cell is started rotating at 500 RPM. When the speed is reached at the setpoint, then the oil drop is injected into the cell. After clicking the measure button, the cell starts measuring the IFTs, displays the plot (IFT vs. Time) on the screen and saves it in the file. The final IFT is taken when the value is stable. 

### 2.4. Rheological Properties Measurements

**Figure 3:**
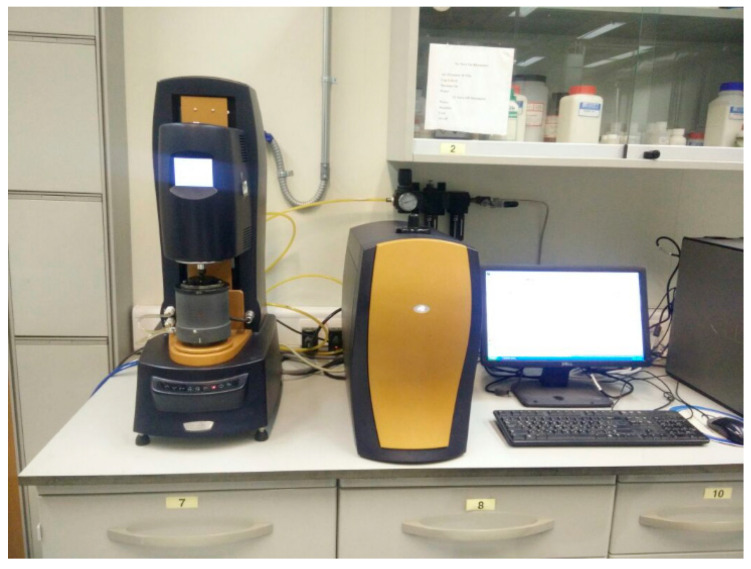
Discovery hybrid rheometer.

The Discovery hybrid rheometer (DHR) was utilized to determine rheological properties of the formulation at wide range of temperatures. The DHR is shown in Figure 3, and it is an advanced combined motor and transducer rheometer consisting of a primary instrument and separate electronics box. First, the geometry (concentric cone and plate) and heating option are selected. And then, enough sample onto the cone is loaded to make sure that when the rotor is lowered. Then the air pressure is adjusted at 30 psi, and to make the rheometer is at zero. After that, the measure is started by clicking the “Start” button, and the results are displayed in the graph and spreadsheet and saved in the specific file. 

Viscosities at different shear rates and temperatures were routinely monitored, and the data obtained when the temperature was stable. Table 2 and Table 3 present the rheological properties of the APG 264, XG, acetone, and butanone.

### 2.5. Core Flooding Experiment

**Figure 4:**
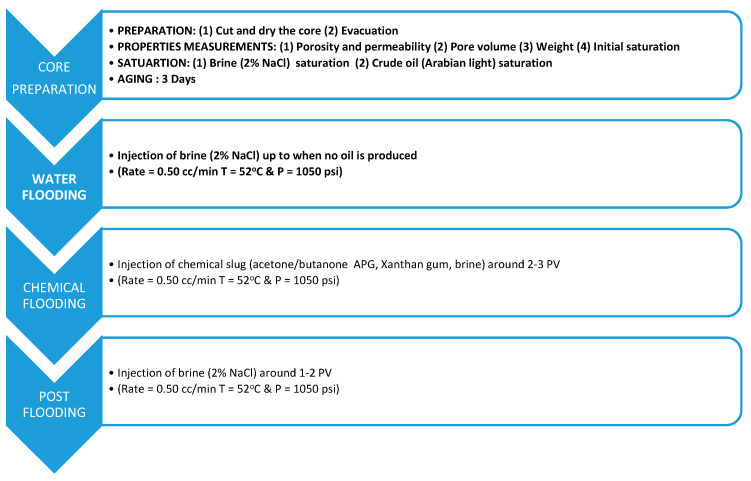
Core flooding procedure.

**Figure 5:**
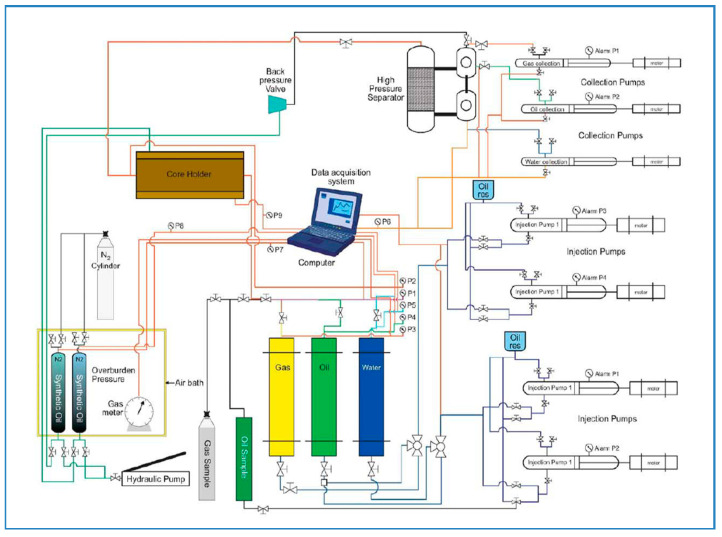
Core flooding experimental setup.

The core flooding process consists of four stages: core preparation, water flood, chemical flood and post-flood. First, two Berea sandstone core plugs were cut and dried at 60 °C in an oven for four hours. The dry weights of the cores were measured, and overburden pressure applied to the core holder. Next, the plugs were placed in a vacuum chamber to remove the air. In the saturation process, brine (2% NaCl) was applied to saturate each core, then flooded with Arabian light crude oil. Next, the oil-saturated cores were put in aging for three days. After aging, the cores were ready for flooding. The cores were first flooded with brine (2% NaCl) when no oil was produced. Next, APG, Xanthan gum, Acetone/Butanone and brine mixture was flowed up to 2–3 PV via post-flooding with brine to ensure no oil remained in the tube and core and collected in the accumulator. Figure 4 demonstrates the core flood process, and Figure 5 describes the experimental setup, respectively. The measured core properties and saturation data are listed in Table 4. The detailed porosity, permeability, and oil saturation calculation and data are given in Table A1, Table A2 and Table A3 in Appendix A. 

### 2.6. Simulation of the Core Flood Experiment

The core flood experiment was simulated using a modified Eclipse simulator [5]. The model was written in a data file (*.data) consisting of sections, a model description, keywords, and comments. The sections included RUNSPEC, GRID, PROS, REGION, SOLUTION, SUMMARY, and SCHEDULE, along with various keywords and comments. 

**Figure 6:**
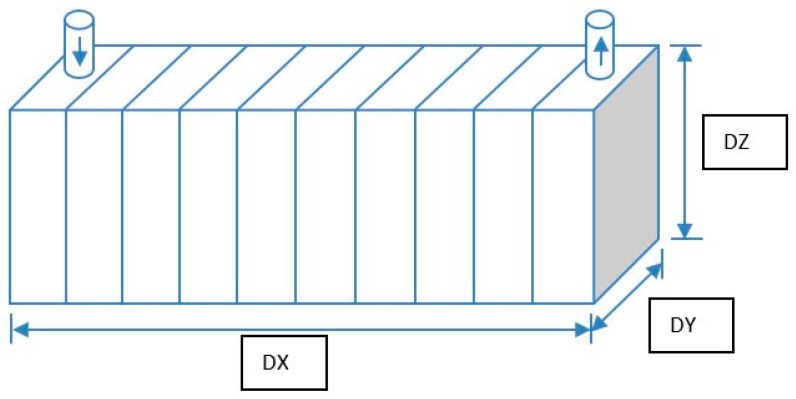
Rectangular core block (10 × 1 × 1).

First, the cylindrical core was converted into a rectangular block (keeping the same volume) and divided into the required number of cells. The injection well was located at one end and the production well at the other. Next, all the necessary data were given in each cell, including the rate. Lastly, the flow equation was linked to all sections and solved from one grid block to the next and within the injection and production wells. A 10 × 1 × 1 rectangular block simulates the sandstone core with APG, Xanthan gum (XG), and a bland acetone formulation. Figure 6 represents the cylindrical core. All input data, descriptions, and keywords are given in Appendix B.

## 3. Results and Discussion

### 3.1. Fluid Properties Measurements

#### 3.1.1. Selection of Green Surfactant

Haq et al. [3] measured the IFT values of four types of APG surfactants at constant concentrations and salinities, keeping the pressure and temperature in the same condition. These were APG 264, APG 8166, APG 8105, and APG 8107. From this non-ionic group, APG 264 offered the best performance based on its IFT reduction capability. APG 264 was thus chosen for the core floods in the present research. 

#### 3.1.2. Selection of Microbial By-Products: Acetone and Butanone

Microbes can produce in-situ bio-surfactants, polymers, alcohols, ketones, acids, and gases in a reservoir. Haq et al. [5] conducted several experiments to examine the combined effects of biosurfactants, APG, and butanol on recovering residual oil. The goal of their study was to develop biobased EOR processes. Such processes are an effective alternative to MEOR methods when MEOR is found to be slow and inefficient. As a continuation of that work, acetone and butanone were selected for the present study to determine the effects of a green surfactant and polymer solution on recovering oil. 

#### 3.1.3. Optimum Concentration of Alkyl Polyglucoside 264

Haq et al. [5] performed a series of IFT measurements to determine the optimum surfactant concentration for core flood experiments. The APG concentrations ranged from 0.20% to 2.00% with brine (2% NaCl). The optimum concentration selected was 0.50% for core flood experimentation. The same concentration was used in the present study. 

#### 3.1.4. Optimum Combined Concentration of Alkyl Polyglucoside 264 and Acetone

**Figure 7:**
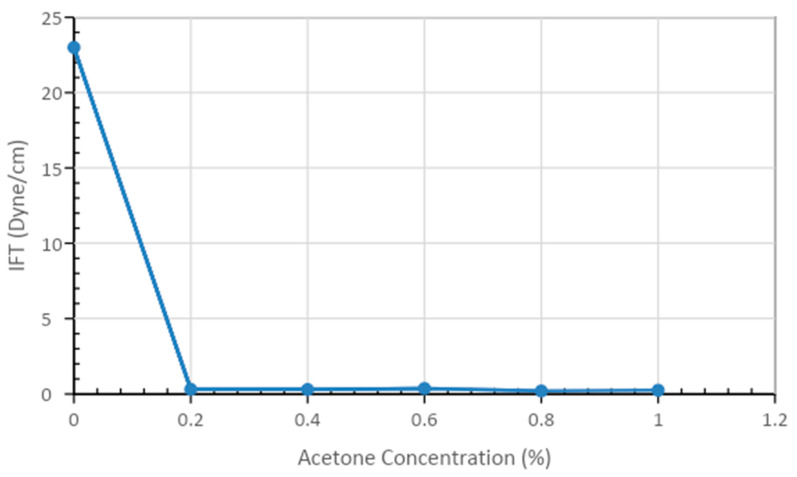
Interfacial tension values for the acetone at concentrations ranging from 0.20% to 1.00% with constant APG (0.50%) and brine (2% NaCl) levels at 52 °C and atmospheric pressure.

The 0.50% APG 264 with acetone concentrations ranging from 0.00% to 1.00% are illustrated in Figure 7 and Table 5. The IFT declined sharply, approximately 22 dyne/cm from 23.00 dyne/cm to 0.20 dyne/cm, and then remained around 0.25 dyne/cm. The optimum concentration of acetone was determined to be 0.60%.

#### 3.1.5. Combined Optimum Concentration of Alkyl Polyglucoside and Butanone 

**Figure 8:**
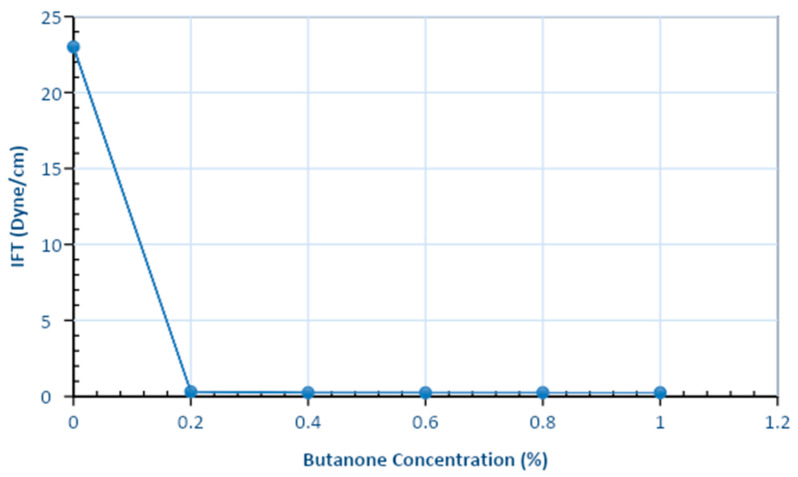
Interfacial tension values for butanone at concentrations ranging from 0.20% to 1.00% with constant APG (0.50%) and brine (2% NaCl) levels at 52 °C and atmospheric pressure.

A number of experiments were conducted to evaluate the influence of butanone combined with APG 264 on interfacial tension. The APG 264 concentration was 0.50%, and butanone concentrations ranged from 0.10% to 1.00%. The brine concentration was 2% NaCl for all samples. The IFT values are given in Table 6 and plotted against the butanone concentrations in Figure 8. The IFT value was 23 dyne/cm at a zero concentration of butanone. It then dropped to 0.54 dyne/cm at a 0.40% concentration of butanone. The IFT remained almost constant at 0.55 dyne/cm for butanone concentrations between 0.40% and 1.00%. A butanone concentration greater than 0.40% was determined to be preferable because if the concentration is greater than the critical micelle concentration, the formation of the micelle is prolonged [15]. Therefore, the optimum concentration of butanone for the flooding experiment was determined to be 0.60% combined with 0.50% APG.

A comparison of the IFT values for the two formulations appears in Table 7. 

#### 3.1.6. Optimum Combined Concentration of Alkyl Polyglucoside 264, Acetone, and Xanthan 

The primary purpose of adding a polymer to the formulation was to boost viscosity and oil recovery and minimize viscous fingering. Three biopolymers have good EOR properties. These are Sclerogucan, Chizophyllan and XG, and all of them are environmentally friendly. Among them, XG is the cheapest [16]. For this reason, XG is selected for this present work.

Said (2018) studied biopolymer concentrations, modifying XG for high pressure, temperature, and salinity reservoirs. A wide range of concentrations was taken for the modification. It was determined that low concentrations of XG produced high-quality polymers. Based on that work, 1000 ppm XG was selected for the present research. Rheological properties are essential when a polymer is added to the solution. Thus, rheological properties measurements for both formulations were performed and are included in this study. 

### 3.2. Rheological Properties Measurements

#### 3.2.1. Influence of Temperature on Viscosity in an SP and Acetone Mixture

**Figure 9:**
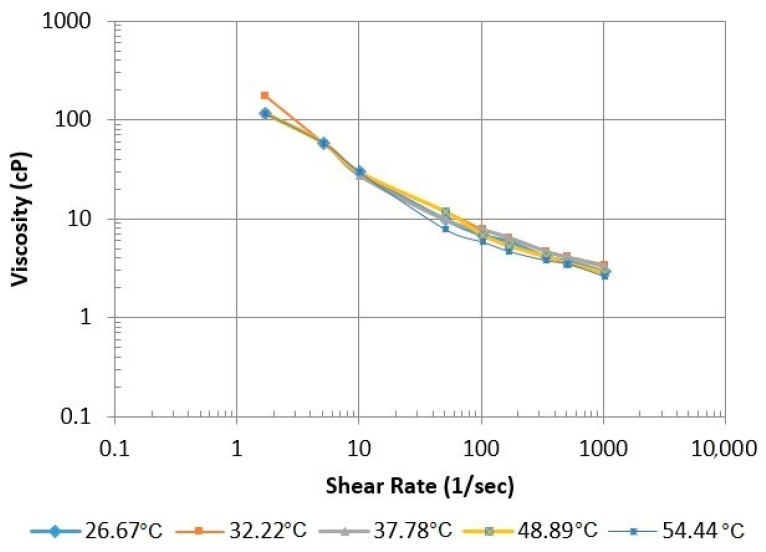
Effects of temperature on the viscosity of the SP and acetone mixture.

The thermal stability of the polymer is a vital parameter for successful EOR application. Therefore, the polymer’s stability at reservoir temperature must be examined before application in the reservoir or core flood experiments. Changes in XG viscosity were investigated by varying the temperature at different shear rates. The temperature was varied from 26.67 °C to 54.44 °C. The results of this experiment are shown in Figure 9, and the data are given in Table 2 in Section 2.4. There was only a minimal change in viscosity with an increase in temperature. Similar patterns were observed in the thermal stability test conducted by Seright and Henrici [17], who ran a series of experiments to determine the thermal stability of XG. The researchers found that it was stable below 60 °C.

#### 3.2.2. Influence of Temperature on the Viscosity of the SP and Butanone Blend

**Figure 10:**
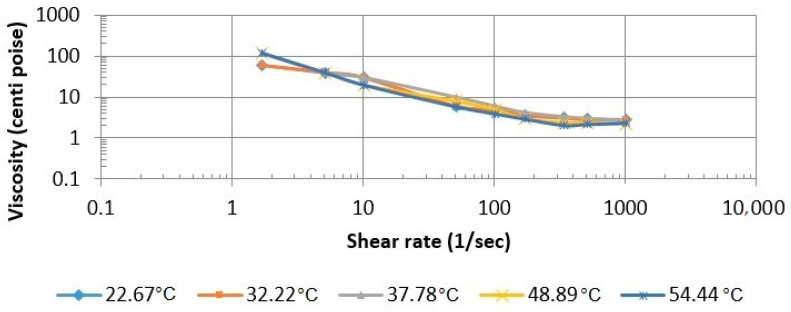
Effects of temperature on the viscosity of the SP and butanone blend.

The influence of temperature variation on the viscosity of the SP and butanone blend was also examined. The viscosity versus shear rate is plotted in Figure 10. The data are given in Table 3 in Section 2.4. The temperatures were varied from 26.67 °C to 54.44 °C. The nature of the plot is similar to a formulation plot. Thus, the temperature was determined to have a negligible influence on viscosity. 

### 3.3. Core Flood Experiment

The oil recovery efficiency of ketones in an SP blend was examined by core flood experiments. This experiment was able to replicate reservoir conditions. Two core floods were performed to explore the oil recovery efficiencies of two ketone and SP combinations: (1) acetone, APG, and XG, and (2) butanone, APG, and XG. Both floods were conducted at 1050 psi and 52 °C

#### 3.3.1. Acetone, APG 264, and Xanthan Gum Formulation

**Figure 11:**
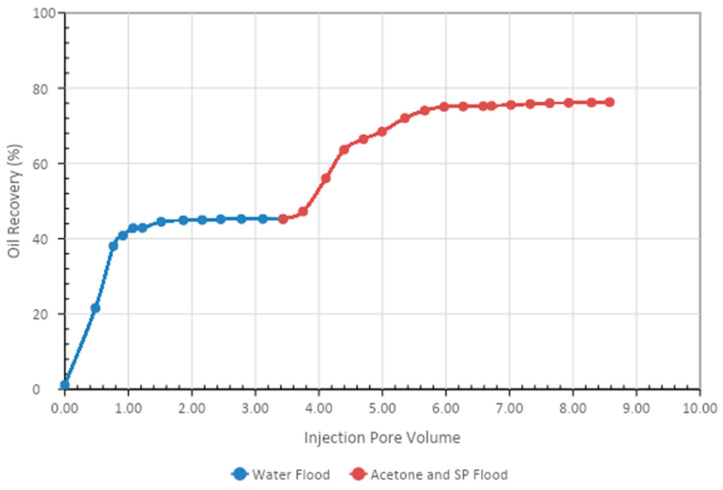
Total oil production from the core after the brine, acetone, and SP blend, followed by a post-flood with brine.

A core flood experiment was performed to observe the oil recovery performance of acetone. Acetone of 0.50% was blended with 0.50% APG and 1000 mg/L (1000 ppm) XG. The results are shown in Table 8 and Figure 11.

First, the core flooding instrument was calibrated with known results. Then, the core was flooded first with brine and then with oil and kept to age for seven days. Next, the test sample was run. In the secondary stage, brine (2% NaCl) was flooded; the oil recovery was 45% of OIIP. In the tertiary stage, an acetone and SP blend was injected. The oil production was 32% after 2.4 pore volumes of injection. The total oil recovery was 76% at the end of the brine, APG slug, and post-flood with the brine process. 

#### 3.3.2. Butanone, APG 264, and Bio-Polymer Formulation

**Figure 12:**
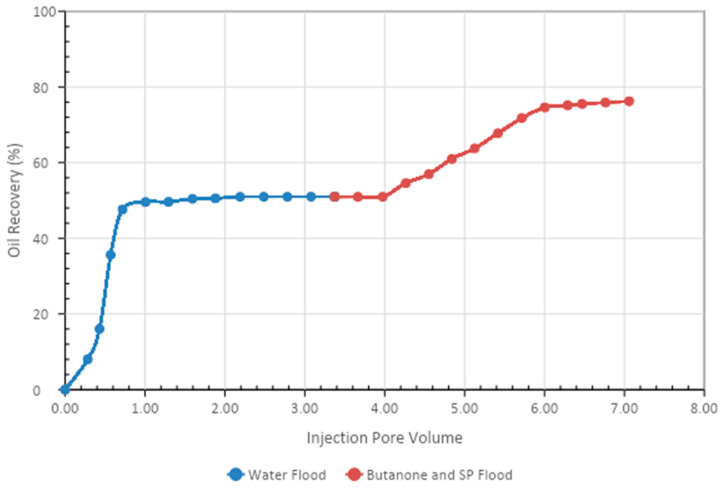
Total oil production from the core after brine, butanone, and SP mixture, followed by a post-flood with brine.

This experiment analyzed the effectiveness of butanone in the SP formulation. The formulation included 0.6% butanone, 0.5% APG 264, and 1000 mg/L XG. The core was vacuumed and saturated with brine (2% NaCl), then flooded with Arabian light crude oil and kept to age for seven days. Next, the core was flooded with brine to recover residual oil saturation prior to the butanone-SP injection. The results of this flood are displayed in Figure 12 and Table 9. At the end of the waterflood, the oil recovery was 50%. Tertiary oil recovery from this formulation amounted to 25.24%. This was a significant amount of additional oil recovery. The total oil recovery of this formulation was 76.22% of OIIP. 

##### 3.3.3. Comparison of the Two Formulations

**Figure 13:**
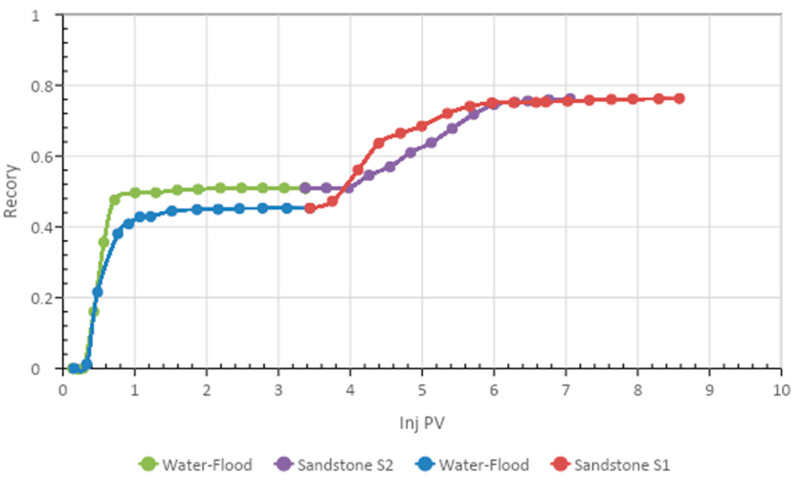
Total oil recovery observed from the two formulations.

**Figure 14:**
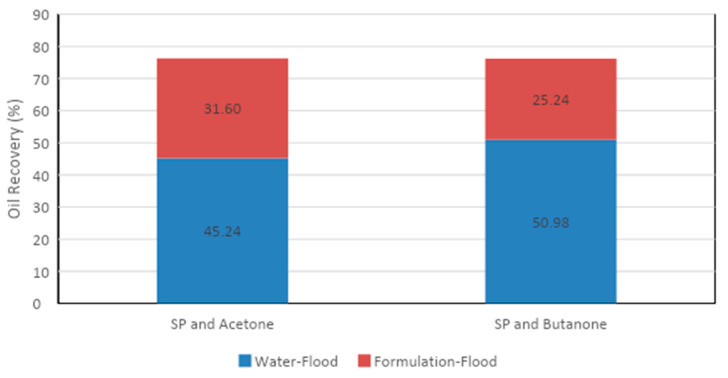
Total oil recovery from the two formulations.

The core flood results for the two formulations are given in Table 10 and Figure 13 and Figure 14. It is clear from Figure 13 that the acetone and SP blend produced 32% tertiary oil. However, the butanone and SP mixture recovered 25% tertiary oil, which was 6 % less than the acetone and SP blend. Therefore, by comparing the two formulations, it can be concluded that the acetone and SP formulation was more efficient at recovering tertiary oil from the sandstone core.

#### 3.4. Simulation of the Core Flooding Experiment

The oil recovery performance of the acetone in the SP formulation was better than that of the butanone. For this reason, this formulation was selected to simulate the core flooding experiment. The results were compared with laboratory data to verify simulation accuracy. Secondary and tertiary recoveries were simulated and matched with the history, and then sensitivity studies were performed. The results are shown in the figures below.

#### 3.4.1. Simulation of the Acetone and SP Blend

**Figure 15:**
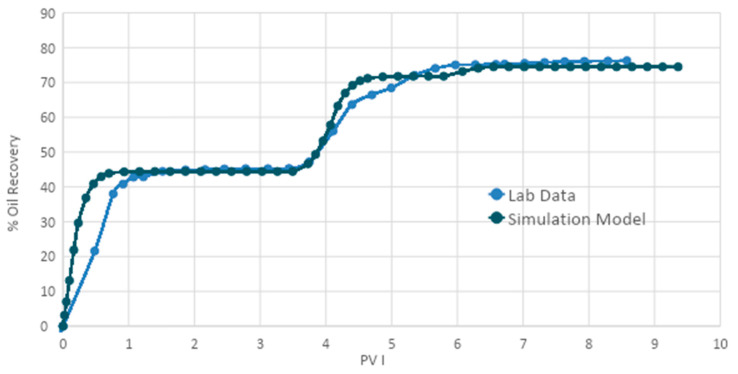
Simulated and laboratory oil recoveries.

In the simulation model, brine (2% NaCl) was injected at a rate of 0.50 cc/min into the core by the injection well until no oil was produced. After that, a solution of 0.6% acetone, 0.5% APG, and 998.96 mg/L (1000 ppm) XG was injected into the core at the tertiary stage. This was followed by a post-flood with brine. All input data are given in Table A4, Table A5, Table A6, Table A7 and Table A8. Figure 15 demonstrates the oil recoveries from the simulation and experiment. The secondary oil recovery in the simulation was 45%. Although the simulation results were the same as the laboratory value, the simulated oil recovery did not match well with the laboratory values between 0.20 PV and 0.80 PV. This was due to improper adjustment of the inputs. However, the simulated tertiary oil curve matched well with the laboratory oil. 

#### 3.4.2. Effect of Adsorption on Oil Recovery

**Figure 16:**
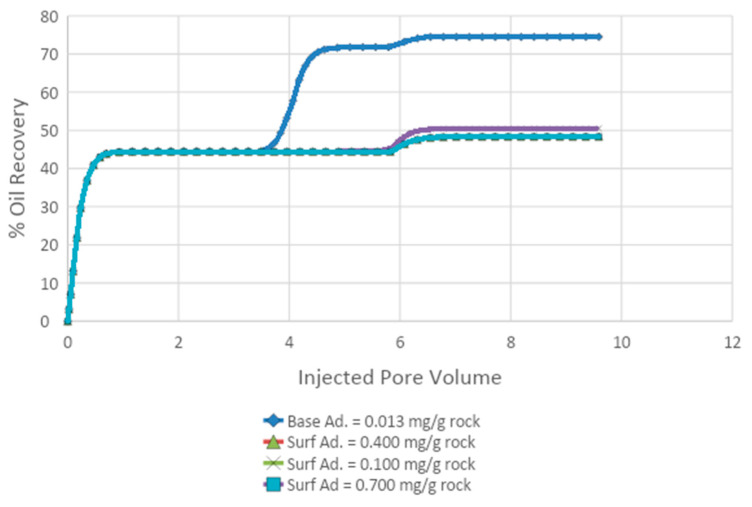
Tertiary oil recoveries at various adsorption levels.

A solution of 0.6% acetone, 0.5% APG, and 1000 ppm XG was injected at a rate of 0.50 cc/min into the model core after water flooding to study the influence of adsorption oil recovery. The base case adsorption was calculated via a history-matching technique to be 0.013 mg/g of rock. The calculation procedure is given in Appendix B. The adsorption values were increased to 0.100, 0.400, and 0.700 mg/mg of rock. The input data appear in Table A4, Table A5, Table A6, Table A7 and Table A8. The oil recoveries with various adsorptions are shown in Figure 16 and Table 11. Oil production declined from 31% to 3%, with an increase in adsorption from 0.013 to 0.40 mg/mg of rock. Thus, there was a substantial reduction in oil recovery with the increase in adsorption due to the adsorption of surfactant by the rock.

## 4. Conclusions

1. The interfacial tensions between the formulations and Arabian light crude oil were determined to identify the optimum concentration of a formulation for core flooding. It was found that concentrations of acetone after 0.60% remained stable, and thus that value was chosen for the core flood experiment. The concentration selected for this formulation was 0.60% acetone, 0.50% APG, and 1000 mg/L (ppm) XG. Similarly, the optimum concentration of the butanone and SP blend was determined to be 0.60% butanone, 0.50% APG, and 1000 mg/L (ppm) XG.
2. As the temperature increased from 33 °C to 57 °C, thermal stability tests established that the formulation of acetone, APG, and XG displayed a steady viscosity.
3. The flooding test confirmed that a concentration of 0.60% acetone, 0.50% APG, and 1000 mg/L XG could recover 31% of the residual oil from a sandstone core. Blending butanone (0.60%) with a surfactant (0.50% APG) and polymer (1000 mg/L XG) gave approximately 25% incremental oil recovery. Acetone was capable of recovering more additional oil than was butanone. The acetone-SP blend was more efficient in terms of recovering additional oil than was the butanone-SP mixture.
4. The simulated oil production from the blended acetone, APG, and XG solution was compared with the experimental values, and the two were found to match reasonably well. The residual oil and total production from the core model were 30% and 75%, respectively.
5. A core flooding experiment on a Berea sandstone core using acetone, APG, and XG mixture was simulated to observe adsorption and injection rate influences on oil recovery. The adsorption results revealed that there was a substantial reduction in oil recovery with an increase in adsorption. However, the rate sensitivity analysis demonstrated that the rate rise had a reverse impact on oil recovery.

## 5. Recommendations

1. Wettability alteration is a vital parameter for designing a suitable surfactant system and predicting oil recovery during surfactant slug injection. This parameter requires thorough examination for both carbonate and sandstone cores [18].
2. It is recommended that endpoint permeability alterations during flooding be investigated. A thorough understanding of these changes would facilitate the precise simulation of mobilized oil flow in cores and the prediction of oil recovery.
3. Further research is needed to examine the effectiveness of the formulation at high temperature and salinity carbonate reservoirs.
4. It recommended to measure the IFT of the two formulations to get a clear understanding of butanone and acetone in EOR.
5. A core flood with only APG and Xanthan gum should be conducted to understand ketone’s effect in oil recovery better.

## Figures and Tables

**Table 1:** Physicochemical properties of acetone and butanone.

Properties	Acetone	Butanone
Chemical Formula	CH_3_–CO–CH_3_	CH_3_–CO–CH_2_CH_3_
Molar mass	58.08 g/mol	72.117 g/mol
Appearance	Colourless liquid	Colourless liquid
Density	0.78 g/cm^3^ at 25 °C	0.80 g/cm^3^ at 25 °C
Boiling point	56.05 °C	79.64 °C
Viscosity	0.29 cP at 25 °C	0.43 cP at 25 °C

**Table 2:** Observed and Measure Data for Acetone (0.6%), APG (0.5%), and Xanthan Gum at Various Temperatures.

Shear Rate (1/S)	Viscosity (cP)				
	26.67 °C	32.22 °C	37.78 °C	48.89 °C	54.44 °C
1021.38	2.937	3.427	3.329	2.839	2.643
510.69	3.72	4.112	4.112	3.525	3.525
340.46	4.112	4.7	4.7	4.112	3.818
170.23	5.874	6.462	6.462	5.287	4.7
102.138	6.853	7.833	7.833	6.853	5.874
51.069	9.791	11.749	9.791	11.749	7.833
10.214	29.372	29.372	27.279	29.372	29.372
5.107	58.744	58.744	58.744	58.744	58.744
1.702	117.488	176.232	117.488	117.488	117.488

**Table 3:** Observed and Measured Data for Butanone (0.6%), APG (0.5%), and Xanthan Gum at Various Temperatures.

Shear Rate (1/S)	Viscosity (cP)				
	26.67 °C	32.22 °C	37.78 °C	48.89 °C	54.44 °C
1021.38	2.839	2.741	2.55	2.154	2.35
510.69	2.937	2.741	2.94	2.35	2.154
340.46	3.231	2.937	3.23	2.35	2.056
170.23	3.525	3.525	4.11	2.937	2.937
102.138	4.895	4.895	5.87	4.895	3.916
51.069	5.874	5.874	9.79	7.833	5.874
10.214	29.372	29.372	29.4	19.581	19.581
5.107	39.163	39.163	39.2	39.163	39.581
1.702	58.744	58.744	117	117.488	117.488

**Table 4:** Core Properties.

Core Plug	Length (cm)	Diameter (cm)	Pore Volume (CC)	Dry Weight (gm)	Porosity (%)	Permeability (mD) Ka	Initial Oil Saturation (%)
1	15.01	3.79	33.78	355.19	19.94	140.31	73.53
2	15.14	3.79	34.88	356.37	20.32	140.31	71.67

**Table 5:** Interfacial Tension Values for Solutions of Acetone (0.00%-1.00%), 0.50% APG, and 2% NaCl.

Formulations	Interfacial Tension (dyne/cm)
Base case	23
APG 0.50% +Acetone 0.1 %	0.31
APG 0.50% +Acetone 0.2 %	0.30
APG 0.50% + Acetone 0.4 %	0.37
APG 0.50% + Acetone 0.6 %	0.20
APG 0.50% + Acetone 0.8 %	0.25
APG 0.50% + Acetone 1.0 %	0.25

**Table 6:** Interfacial Tension Values for APG 264 and 0.5% Butanone at Concentrations Ranging from 0.1% to 1.0%.

Formulations	Interfacial Tension (dyne/cm)
Base case	23
APG 0.50% + Butanone 0.10%	0.31
APG 0.50% + Butanone 0.20%	0.25
APG 0.50% + Butanone 0.40%	0.24
APG 0.50% + Butanone 0.60%	0.25
APG 0.50% + Butanone 0.80%	0.25
APG 0.50% + Butanone 1.00%	0.25

**Table 7:** IFT Comparison for the Two Formulations.

	IFT	
Concentration	Acetone	Butanone
0	23	23
0.1	0.31	0.31
0.2	0.30	0.25
0.4	0.37	0.24
0.6	0.20	0.25
0.8	0.25	0.25
1	0.25	0.25

**Table 8:** Flood Results for Acetone, APG 264, and Xanthan Gum Blend.

Core	PV (cc)	Oil Volume (cc)	Soi (%)	Water Flood Recovery		S.P. Flood Recovery		Total
				cc	%	cc	%	%
S1	34	25	73.5	11.31	45	7.755	31.6	76.26

**Table 9:** Flood Results for Butanone, APG 264, and Xanthan Gum Mixture.

Formulation 2: 0.5% APG + 0.6% Butanone + 1000 mg/L XG + 2% NaCl water								
Core	PV (cc)	Oil Volume (cc)	Soi (%)	Water Flood Recovery		S.P. Flood Recovery		Total
				cc	%	cc	%	%
S2	34.88	25	71.67	12.75	50.98	7.11	25.24	76.22

**Table 10:** Summary of the Two Formulations.

Formulation	Core	PV (cc)	Oil Volume (cc)	Soi (%)	Water Flood Recovery		S.P. Flood Recovery		Total
					cc	%	cc	%	%
SP and Acetone	S1	34	25	73.5	11.31	45	7.755	31.6	76.26
SP and Butanone	S2	34.88	25	71.67	12.75	50.98	7.11	25.24	76.22

**Table 11:** Effects of Adsorption on Oil Recovery.

Adsorption (mg/g rock)	Oil Recovery (%) at 8 PV
Base—0.013	75
Low—0.100	50
Medium—0.400	48
High—0.700	48

**Figure A1:**
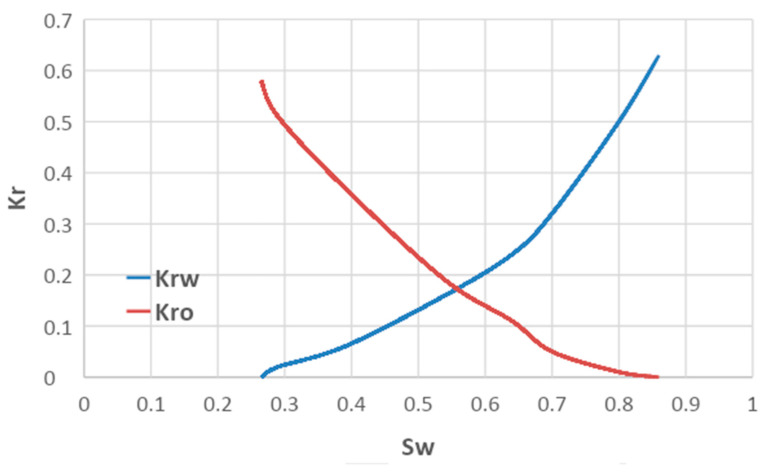
Relative permeability and water saturation plot for a sandstone core sample.

**Figure A2:**
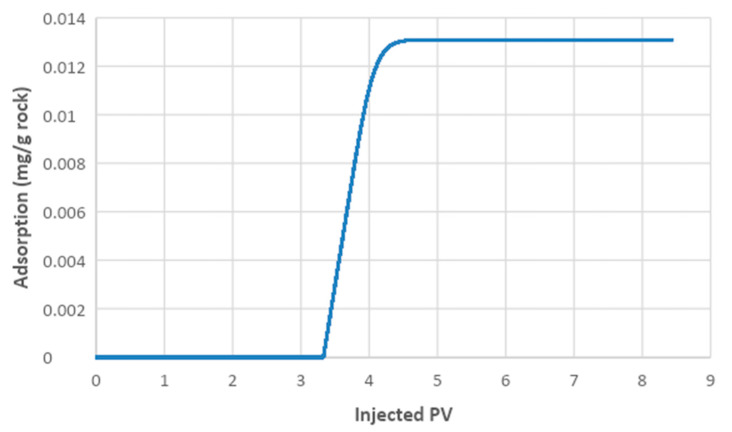
Amount of adsorption as a function of the injected pore volume.

## Data Availability

Not applicable.

## Nomenclature

APG	Alkyl Polyglucosides
CMC	Critical Miscible Concentration
EOR	Enhanced Oil Recovery
HLB	Hydrophilic/Lipophilic Balance
GEOR	Green Enhanced Oil Recovery
IFT	Interfacial Tension
MEOR	microbial enhanced oil recovery
OIIP	Oil Initially In Place
TOR	Tertiary Oil Recovery

## Appendix A. Core Flooding Experiments

### Appendix A.1. Procedure

#### Appendix A.1.1. Water Saturation

Each core is placed in a cell vacuumed while the core is inside. Then, the water is sucked into the cell using the vacuum. Once the cell is filled with water, a pump is used to increase the pressure inside to 2500 psi, which forces the water to enter the core to be saturated. The water used is 2% NaCl, and the saturation process is left to continue for one day at a minimum.

#### Appendix A.1.2. Porosity Calculation

When the water saturation is complete, the weight is measured again to determine the weight difference between the dry and saturated cores. This gives the weight of the water inside. Knowing the weight and density of the water inside gives the volume of the water inside the core, which can be used along with the bulk volume of the core to obtain the porosity of the core. Table A1 shows the porosity calculation for each core.

#### Appendix A.1.3. Permeability Determination

A permeability test is conducted using the core flooding machine. The core will stay inside until the end of the core flooding experiment.

After saturating and weighing the core, it is placed in the core flooding machine. This machine is used to flood water through the core at different flow rates (the same water with which the core was saturated). This will give additional pressure drops. From Darcy’s equation, the permeability of the core can then be measured. Table A2 shows the permeability measurements obtained for each core.

#### Appendix A.1.4. Oil Saturation

When the permeability test is complete, the flood is switched from water to oil. In this project, the oil was Arabian light. To get the best oil saturation results, the oil can flood what equals three pore volumes. This ensures that all of the moveable water has been moved. In addition, the outflow is collected once the switch from water to oil happens. This helps track how much oil is pumped through the core. More importantly, this helps determine the water and oil saturation by how much water has left the core compared to the previous volume of water inside. Table A3 shows the oil saturation results.

**Table A1:** Porosity Calculation.

Core	Weight Dry (g)	Length (cm)	Diameter (cm)	Total Volume (cc)	Weight Saturated (g)	Pore Volume (cc)	Porosity (%)
S1	355.19	15.01	3.79	169.32	389.34	33.78	19.94
S2	356.37	15.14	3.8	171.62	391.67	34.88	20.32

**Table A2:** Average Permeability Determination.

Core		1.00	2.00	3.00	4.00	5.00
Sandstone S1-S2	dp (psi)	5.10	2.20	1.20	0.60	0.00
	dp (atm)	0.35	0.15	0.08	0.04	0.00
	q (cc/min)	4.00	2.00	1.00	0.50	0.00
	q (cc/s)	0.07	0.03	0.02	0.01	0.00
	Permeability (mD)	140.31				

**Table A3:** Oil Saturation.

Core	Total PV (cc)	Water V (cc)	Oil V (cc)	Soi (%)	Swi (%)
S1	34	9	25	73.53	26.47
S2	34.88	9.88	25	71.67	28.33

## Appendix B

### Appendix B.1. Model Description

The Eclipse model was developed to describe the core sample using keywords and comments employed to enhance readability. The data file is sectioned into RUNSPEC, GRID, PROS, REGION, SOLUTION, SUMMARY, and SCHEDULE, with various keywords for entering particular sections. The RUNSPEC section allocates memory, specifies the general settings of the simulation, and defines the type of model being created. The GRID section stipulates the grid geometry and properties, including porosity, permeability, and grid sizes and dimensions. The rock and fluid properties are entered in table form in the PROPS section, and the REGION section subdivides the model. Fluid contacts and equilibration data are entered in the SOLUTION section, used to initialize the model. Finally, the SCHEDULE section is where the wells are defined and well control and time steps stated. Output from the simulation is specified in the SUMMARY section.

### Appendix B.2. Grid Model

The grid block below shows that the simulation comprises a single injector well and a single producer well placed at the tail end of the grid block. This ensures an efficient sweep during the water flooding and enhancement stages. The simulation model was developed based on three dimensions, with the geological layers having 10 columns of cells in the lateral direction and 1 column each in the transverse direction. Laboratory measurements were done on a cylindrical core sample since it best represented the shape of the reservoir. However, a rectangular core was assumed for the simulation. For better comparison, the cylindrical core sample and rectangular core of the sandstone core sample were supposed to have the same volumes, based on the following deduction. The grid model with the dimensions used for this research is given in Figure 1.

The mathematical evidence showing that the cylindrical and rectangular cores have the same volume is given below:

Core length = 15.08 cm

Diameter = 3.8 cm

Cylindrical core:

$$\mathrm{Cross}-\sec\mathrm{tional}\;\mathrm{area}=\Pi D^{2}/4=\frac{\pi \times 3.8^{2}}{4}=11.34\;{\mathrm{cm}}^{2}=\left(3.36\;\mathrm{cm}\times 3.36\;\mathrm{cm}\right)\;\mathrm{Volume}=\Pi D^{2}/4\times L=11.34\times 15.08=171\;{\mathrm{cm}}^{3}\;$$

Rectangular core:

DY = DZ = 3.36 cm

Length = DX = 15.08 cm

$$\mathrm{Cross}-\sec\mathrm{tional}\;\mathrm{area}=\mathrm{DY}\;\times \;\mathrm{DZ}=3.36\;\mathrm{cm}\times 3.36\;\mathrm{cm}=11.34\;{\mathrm{cm}}^{2}\;\;\;\mathrm{Volume}=\mathrm{DY}\;\times \;\mathrm{DZ}\;\times \;L=\mathrm{DY}\;\times \;\mathrm{DZ}\;\times \;\mathrm{DX}=3.36\;\mathrm{cm}\times 3.36\;\mathrm{cm}\times 15.08=171\;{\mathrm{cm}}^{3}\;$$

### Appendix B.3. Input Parameters

The input used for this simulation study was comprised of primary core data and properties, reservoir and simulation properties, relative permeability and saturation levels, fluid properties, and interfacial tension values for different concentrations. Table A4 below includes the primary sandstone core data and properties. Other input parameters are described under a separate heading.

**Table A4:** Core Data and Properties.

Core Plug Sandstone	Length (in)	Diameter (in)	Pore Volume (cc)	Dry Weight (gm)	Porosity (%)	Permeability (Absolute) (mD)
1	5.911	1.492	34	389	19.94	138

### Appendix B.4. Reservoir and Simulation Model Properties

This section contains the input properties depicting the nature of the reservoir and conceptualizes the rectangular cube model used in the simulator to represent the cylindrical core sample in the lab. Table A5 shows that the reservoir had a kv/kh value of 1, since both the horizontal and vertical permeability values were equal. The OWC was 690 m with a datum of 680 m. The pressure at this datum was given as 1050 psi.

**Table A5:** Reservoir and Simulation Model Properties.

Parameters	Values
Model physical dimension	15.08 cm × 3. 36 cm × 3.36 cm
Datum pressure	72.41 bar = 1050 psi
Datum depth	680 m
Depth at oil-water contact	690 m
Porosity, Φ	19.94
Horizontal permeability, k_h_	Top layer: 138 mD
Vertical permeability, k_v_	Top layer: 138 mD
Well/tube diameter (mm)	4.00

### Appendix B.5. Relative Permeability and Saturation

This is one of the most vital input parameters when it comes to reservoir simulation. Relative permeability is defined as the ratio of effective permeability of a fluid at a given saturation to the base permeability. The base permeability can be absolute, air, or the effective permeability of the non-wetting phase at initial water saturation. When the relative permeability is 1, it means the base permeability is the effective permeability. Relative permeability combined with absolute or intrinsic permeability reflects the flow capacity for a fluid phase at a given pressure differential across a core (Richard 2017). Measuring relative permeability requires a strongly coupled effort combining experimental techniques and data reduction skills. Some of these practical approaches are challenging to implement. Usually, they have more straightforward data reduction procedures, while those less challenging to perform often require detailed data reduction procedures. In convention reservoirs, relative permeability can be determined through laboratory, analogy, or modelled correlations (Roychaudhuri et al. 2013).

In this research, the SOI was given as 73.5%. This became the basis for generating the relative permeability values used in the simulation. Table A6 shows the input parameters for the relative permeability. Figure A1 is the plot of the sandstone core sample.

**Table A6:** Relative Permeabilities and Saturations.

Sw	Krw	So	kro
0.265	0	0.735	0.58
0.29	0.02	0.71	0.51
0.39	0.06	0.61	0.37
0.54	0.16	0.46	0.19
0.64	0.24	0.37	0.11
0.7	0.32	0.3	0.05
0.8	0.5	0.2	0.01
0.86	0.63	0.14	0

### Appendix B.6. Fluid Property Data

This section contains the input properties for the formulations used. It is important to note that before the history match was done, Table A7 and Table A8 were used as the initial input properties for the formulation and simulation, respectively.

**Table A7:** Formulation Fluid Properties.

Parameters	Values
Oil density (kg/m^3^)	936
Water density (kg/m^3^)	1000
Oil Viscosity (cP)	0.47
Water Viscosity (cP)	0.34
Adsorption function (mg/g rock)	0.013
Initial IFT (dyne/cm)	23
Final IFT (dyne/cm)	0.25
Well/tube diameter (mm)	4.00
Liquid rate (cc/min)	0.50
Volumetric sweep efficiency	1

**Table A8:** IFT Values for Different Surfactant Concentrations.

Surfactant Concentration (%)	Acetone Concentration (%)	Total Concentration (%)	IFT (dyne/cm)
0.5%	0	0	23.00
0.5%	0.2	0.7	0.31
0.5%	0.4	0.9	0.30
0.5%	0.6	1.1	0.37
0.5%	0.8	1.3	0.20
0.5%	1	1.5	0.25

### Appendix B.7. Adsorption Determination

The simulator determined the base adsorption. Two keywords, SURFADS and FTADSUR, were added to the summary section of the Eclipse datafile. The simulation was run after adding all necessary lab data to the input file. The Eclipse simulator uses Equation (A1) to calculate the surfactant adsorption, which is a function of the surfactant concentration. The surfactant adsorption calculated concerning the injected pore volume is plotted in Figure A2. The base adsorption was selected based on a history-matching technique. Based on the simulation, the quantity of adsorbed surfactant on the rock surface was 0.013 mg/g rock.

(A1) $$M_{AS}=PV_{cell}\times \frac{1-\emptyset }{\emptyset }\times \rho_{mass}\times CA\left(C_{surf}\right)$$

where *M*_AS_ = the mass of the adsorbed surfactant, *PVcell* = the pore volume of the cell, *Ø* = the porosity, *ρ_mass_* = the mass density of the rock in the SURFROCK keyword, and *CA*(*C_surf_*) = the adsorption isotherm as a function of the local surfactant concentration in the solution.
